# Exposure to a common urban pollutant affects the survival and swimming behaviour of creek chub (*Semotilus atromaculatus*)

**DOI:** 10.1111/jfb.14685

**Published:** 2021-02-15

**Authors:** Christopher M. Bunt, Bailey Jacobson

**Affiliations:** ^1^ Biotactic Fisheries Research and Monitoring Biotactic Inc. Kitchener Ontario Canada

**Keywords:** creek chub, dissolved oxygen, pet waste, physiological stress, urban pollution

## Abstract

Anthropogenic effects on the aquatic environment are ever present and ever increasing and while a plethora of aquatic contaminants are known to affect fishes, one ubiquitous and increasingly prevalent world‐wide urban runoff pollutant is frequently disregarded, and that is pet waste. While dog waste has been identified as a significant factor contributing to bacteria and nutrient loading within receiving waters and the associated water quality changes are known to affect fishes, the impact of uncollected dog faeces on urban fish populations has never been directly investigated. In this study we exposed creek chub (*Semotilus atromaculatus*), a widespread tolerant stream minnow, to various realistic concentrations of dog waste as simulated urban park runoff testing both fresh and dried dog faeces in both stagnant and aerated water for 96 h to investigate the impact on fish survival and behaviour. Creek chub percentage mortality increased significantly relative to controls and across an exposure gradient and was likely caused by anoxic conditions. Survivors were initially smaller while those that died were initially larger and presented with abnormal abdominal subdermal lesions post‐exposure. Additional indicators of physiological stress included significantly increased rates of aquatic surface respiration and changes in flume test derived swimming motivation metrics with increased exposure concentrations. Both mortality and behavioural responses were alleviated by aeration. Furthermore, trials with fresh and dried faeces differed only in time‐to‐death and swimming metrics where results from dried trials were similar to those from aerated experiments. Results demonstrated the impact that the global dog waste management problem can have on aquatic communities with effects on creek chub likely to be more severe for less pollution‐tolerant species and also likely to be exacerbated under future scenarios that consider climate change and increased urbanization.

## 1 INTRODUCTION

Anthropogenic effects on the aquatic environment are ever present and ever increasing with habitat destruction and fragmentation, releases of exotic species, and industrial, municipal and agricultural run‐off all consequences of human actions and inaction. The negative impacts of human activities on aquatic populations are easily observed. Construction increases rates of shoreline erosion, dams disconnect rivers and impede fish migration, non‐native species outcompete native, and agricultural, municipal and industrial contaminants such as polychlorinated biphenyls (PCBs) and dichlorodiphenyltrichloroethane (DDT) can cause changes in fish behaviour and growth (sublethal) or even mortality (Little & Finger, 1990; Melvin & Wilson, 2013), reducing fish community diversity and altering population structure (Henshel *et al*., 2005; Simon *et al*., 2013). While a plethora of aquatic contaminants are known to affect fishes, one frequently disregarded major environmental pollutant that many humans may not realize should also be classified as such is pet waste. This ubiquitous and increasingly prevalent urban pollutant has the unrecognized potential to impact the health of fish populations worldwide.

As the human population continues to increase and urbanize, so too does the pet population and at an even greater rate, with the number of registered pet dogs in the United States increasing from 70 million in 2012 to 76.8 million in 2017 (American Veterinary Medical Association, 2018) and the number in Canada increasing from 7.6 million in 2016 to 8.2 million in 2018 (Canadian Animal Health Institute, 2019). The average dog produces 100–200 g of faeces/day and the average gram of faeces contains 2.3×10^7^ faecal coliforms. It is thus unsurprising that often up to 30% of total measured bacteria within urban receiving waters (*e.g*., municipal drains, storm water ponds, streams) has been directly attributed to dog inputs (Ellis, 2004; O'Keefe *et al*., 2005). In fact dogs are now often identified as the single greatest contributor of faecal coliform loading within urban areas (Hobbie *et al*., 2017; Selvakumar & Borst, 2006), with even small amounts of faecal bacteria found to significantly reduce water quality (Ervin *et al*., 2014). For example, Ervin *et al*. (2014) studied the origin of faecal indicator bacteria sampled within the surface water of a coastal beach in Santa Barbara County, California, and found that an input of only 24 g of dog faeces/day (a single elimination by a small dog) into an upstream creek was enough to raise bacterial levels in both a downstream lagoon and subsequently the beach surf zone.

Not only do inputs of dog faeces increase bacteria levels and thus biochemical oxygen demand (BOD; Penn *et al*., 2009), but other associated changes in water quality may also affect fish communities. Dog waste has been found to be a significant source of nutrient loading, accounting for up to 76% of total phosphorus and 28% of total nitrogen levels in some urban watersheds, which is more than is linked to agricultural practices (Hobbie *et al*., 2017). Nutrient loading, in turn, can lead to eutrophication and the production of algal masses that shift towards blue‐green algae blooms (Hobbie *et al*., 2017; Smith *et al*., 1999). Such increased production can translate into an overload of organic carbon, causing reductions in dissolved oxygen (DO) and increases in pH (Dodds & Welch, 1999). Reductions in the amount of DO available to fish can also occur as a result of the ammonia contained within dog waste itself as ammonia is converted to nitrite and nitrate by *Nitrosomonas* and *Nitrobacter* bacteria spp. (Lewis & Morris, 1986) through a process that directly consumes oxygen. Nitrite also oxidizes the iron in haemoglobin, converting it to methaemoglobin in fish, a form which cannot carry oxygen and reduces blood oxygen carrying capacity, potentially leading to anoxia (Lewis & Morris, 1986; Russo *et al*., 1974). Changes in DO (hypoxia) resulting from nutrient loading, increased ammonia and nitrate/nitrite, and BOD can influence fish behaviour, growth and mortality (Dodds & Welch, 1999; Lewis & Morris, 1986; Russo *et al*., 1974). Indeed all of the above have been associated with the presence of degraded fish communities and negatively correlated with response metrics such as biotic integrity, particularly within small or channelized systems (Meador & Goldstein, 2003; Miltner & Rankin, 1998; Smiley Jr. *et al*., 2009) that are often found within urbanized areas (Pollock *et al*., 2007).

Despite the clear theoretical link that dog waste affects water quality and water quality affects fishes, the impact of dog faeces on urban fish populations has never been directly studied. This is surprising given the recent flux of studies that indicate dog waste is a significant source of environmental pollution, the long history of studies measuring the effects of various pollutants on numerous fish species and the fact that still only an estimated 60% of Americans pick up their pet waste (Swann, 1999; Waters *et al*., 2011), with fewer actually doing so daily (Serrano & DeLorenzo, 2008). As such, the goal of this study was to examine the direct effects of solid dog waste on fish survival and behaviour. To do this we exposed creek chub *Semotilus atromaculatus* (Mitchill 1818) to various concentrations of dog faeces in addition to alternate states of dog faeces (fresh and dried) and differing water conditions (stagnant and aerated) for a 96 h period. Alternate states were tested to determine if, contrary to common perception, dried seemingly innocuous faecal samples would still elicit a fish response and differing water conditions were tested to better approximate stream as well as pond receiving water characteristics. We expected that fish mortality and behaviour would still be affected by exposure to dried faeces, but to a lesser degree compared to measured responses to exposure to the more virulent fresh samples. We also predicted that no mortality or behavioural shifts would occur during exposure within trials with aeration, since aeration would alleviate any potential faeces‐induced low DO levels. Creek chub were selected for this study as this species is one of the most widespread stream minnows in eastern North America (Dubé *et al*., 2006) and is commonly found in urban creeks and waterways, often in abundance within areas with degraded habitat and poor water quality (Katz & Gaufin, 1953; Reash & Berra, 1987; Simon *et al*., 2013). This suggests that creek chub can tolerate poor water quality and thus serve as an umbrella species for this study with any measured responses likely to also extend to, and be more acute for, a wide range of less tolerant fish species.

## 2 MATERIALS AND METHODS

### 2.1 Experimental set‐up

Faecal samples were obtained from a single healthy 6‐year‐old Chihuahua/Yorkshire terrier cross (weight = 6.8 kg, Science Diet pet food). Samples were either used the same morning they were deposited (fresh) or were dried for 24 h in front of a space heater prior to use. Both fresh and dried samples were sent to A&L Canada Laboratories Inc. and analysed for the amount of moisture, nitrogen, phosphorus, potassium, NH4‐N and coliform‐forming units (CFU) in each sample.

**FIGURE 1:**
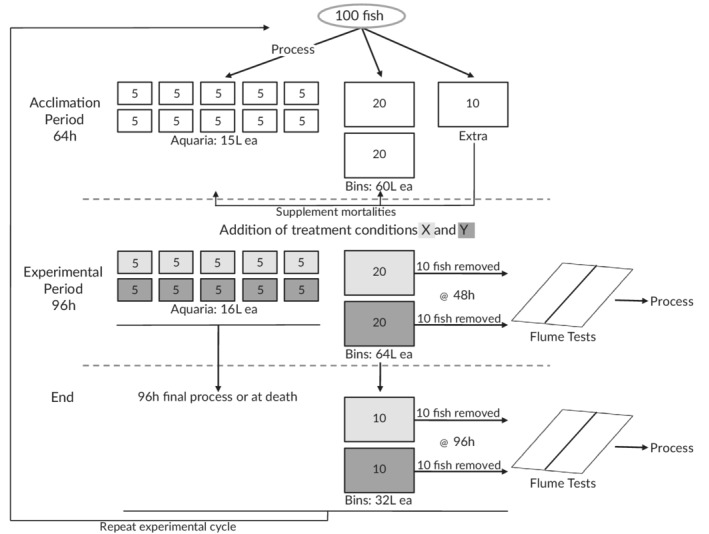
Schematic diagram of experimental set‐up and one complete trial cycle. Treatment conditions X and Y represent two of the seven conducted treatments. Treatments included unaerated fresh faecal samples at zero concentration (control×F), 1×concentration (0.092 g/l, 1×F), 2×concentration (0.184 g/l, 2×F) and 4×concentration (0.368 g/l, 4×F), and desiccated faecal samples at 4×concentration (0.368 g/l, 4×D), aerated fresh faecal samples at zero concentration (control×A) and 4×concentration (0.368 g/l, 4×A)

One hundred adult creek chub were obtained for each experimental cycle from a local bait supplier who captured the fish from moderately urbanized streams within the Grand River watershed (Ontario, Canada) 1–2 days prior to experimental acclimation. Fish were transported to the laboratory and divided among 10 experimental glass aquaria each filled with 15 l of aerated water and two large plastic bins each filled with 60 l of aerated water (Figure 1). Five fish were placed within each aquaria after first being measured (cmTL), weighed (g) and photographed (*i.e*., processed). Without processing, 20 fish were placed in each bin and the remaining 10 fish were held in a cooler with aerated water and were only used to augment aquaria or bin density if mortality occurred during acclimation. Fish were left to acclimate for 64 h.

After acclimation, treatments were conducted two at a time for a period of 96 h. Each treatment utilized five of the 10 aquaria (replicates) wherein fish survival, one of four measured behavioural metrics and water quality were determined. Each treatment also utilized one of the two bins, the fish within which were used in flume swimming trials from which the remaining three behavioural metrics were calculated. At the beginning of each treatment, aeration either was or was not removed and the relevant amount and type of pure dog faeces was weighed out for each aquarium and bin separately. To simulate urban park runoff, portioned faecal samples for each experimental container were individually mixed with a hand blender for 30 s with either 1 l (aquaria) or 4 l (bin) of additional water prior to addition. Note that dry faecal trials were only replicated three times (three aquaria and one bin, total of 35 fish) due to a lack of faeces availability.

Throughout both acclimation and experimental periods, fish were left unfed and exposed to a 14 h:10 h light:dark photoperiod to mimic natural light conditions at the time experiments were conducted (summer–fall). Each aquarium and bin was covered with screening to prevent jumping and isolated by white opaque barriers to reduce the amount of extraneous stress experienced by the fish. All water used throughout each experimental cycle was obtained from the Grand River (Kitchener, Ontario) and was first filtered and screened prior to use. Between experimental cycles all aquaria, bins, screens and aeration tubing were disinfected using ethanol, rinsed and thoroughly dried; new air stones were used for each cycle.

### 2.2 Treatments and water quality

A total of seven treatments were tested over the course of four experimental cycles conducted between July 11 and October 11 2019, the last cycle testing the one final remaining treatment. Five treatments were not aerated and exposed fish to the following conditions: fresh faecal samples at zero concentration (control×F), 1×concentration (0.092 g/l, 1×F), 2×concentration (0.184 g/l, 2×F) and 4×concentration (0.368 g/l, 4×F), and desiccated faecal samples at 4×concentration (0.368 g/l, 4×D). The remaining two treatments were aerated and exposed fish to fresh faecal samples at zero concentration (control×A) and 4×concentration (0.368 g/l, 4×A). The 1×concentration of dog faeces was used to develop a range of concentrations within which a response was expected, but not guaranteed, based on pilot experiments conducted in June 2019. Following similar types of calculations performed in Ervin *et al*. (2014), the calculation of this concentration was based on the maximum amount of *E. coli* found in water samples taken from Calgary dog parks (Tambalo *et al*., 2012) and the minimum amount of *E. coli* from the average piece of dog faeces (Walker & Garfield, 2008). The concentration in g/l of dog faeces required to reach the bacterial levels in the Calgary sample was then calculated and the Calgary derived g/l value was scaled based on the average number of dogs in 10 major cities around the world.

Changes in water quality parameters were monitored daily at ~10 am within each aquarium and bin using a multimeter probe (model HI9829, Hanna Instruments) to measure pH, DO (mg/l), ammonia (mg/l) and temperature (°C). Nitrate (mg/l) was also measured using a standard aquarium test kit.

### 2.3 Survival

Experimental aquaria were regularly monitored for fish mortalities and deceased fish were immediately removed and the time or time range of death recorded. Time of death at night was monitored with an overhead infrared fish monitoring camera system (BRAVO, Biotactic Inc.). On either mortality or the end of the 96 h exposure period fish were re‐measured for length (cmTL) and weight (g), photographed and given a score from 0 to 3 based on the presence and severity of observed epidermal or subdermal blemishes, sores or wounds (final measurements). Bins were also regularly checked for fish mortalities with deceased fish immediately removed without further processing.

### 2.4 Behaviour

Throughout the 96 h exposure interval, three sets of 2 min long visual observations were conducted at each aquarium each day at 9 am, 12 pm and 4 pm to monitor fish air surface breathing or aquatic surface respiration (ASR). ASR percentage as a function of duration and number of fish observed within the 2 min period was calculated to provide a metric indicative of physiological stress.

To further monitor fish behaviour, swimming trials were conducted to determine the impact of exposure on motivation. Trials were performed within a two‐channel flow‐through flume (channel dimensions 1.52×0.20 m) set on a 5% slope with a 5×5 cm gridded floor and supplied with filtered and screened Grand River water. At 48 h post‐treatment exposure up to 10 fish were removed from each bin and transported to a nearby testing area where they were left to recover from transportation for 1 h in fresh aerated water. Afterwards two fish, one fish from each of the two conducted treatments, were tested simultaneously within each flume channel, with the side used for a particular treatment alternating between fish. Fish were first left to acclimate within the flume for 10 min in a downstream partitioned area, after which time the divider was removed and fish were allowed access to the remainder of the channel. Swimming behaviour was video recorded for 10 min (Bonansea *et al*., 2016) and then fish were removed from the channel and measured, weighed and photographed. Videos were later scored for three metrics: (a) time delay to leave the acclimation area (seconds); (b) total time of active movement within the grid area (seconds); and (c) total number of squares at least half of the fish body passed through or distance travelled (Bertram *et al*., 2018; Bonansea *et al*., 2016; Vieira *et al*., 2009). Note that swimming behaviour was also tested at 96 h post‐exposure but since most of the observed effects occurred within the first 48 h, and not all treatments could be tested at 96 h due to bin mortalities, these data have not been included. All trials were conducted between 1100 and 1530 h.

### 2.5 Statistical analyses

Differences between treatments with respect to water quality parameters (other than temperature, which was independent of faecal input) were explored using principal component analysis (PCA) on a correlation matrix of *z*‐score transformed variables. Water quality values used within the PCA were the average daily measurement between 24 and 96 h post‐exposure of each parameter for each aquarium (replicate).

To determine the influence of exposure on the final percentage mortality observed within trials, a series of multinomial or binomial exact goodness‐of‐fit tests were conducted to compare levels across several treatment combinations, including: (a) four concentrations (control, 1×, 2× and 4×F) with *post hoc* binomial pairwise comparisons computed if significant; (b) 4×F and 4×D; (c) control×A and 4×A; (d) control×A and control×F; and (e) 4×A and 4×F treatments. As fish size likely influences physiological tolerance (Lewis & Morris, 1986; Russo *et al*., 1974), the relationship between initial fish mass and trial survival was evaluated using a Mann–Whitney–Wilcoxon test including all replicates across treatments wherein mixed mortality occurred. To ensure that any differences between treatment outcomes were not an artefact of chance variation in initial fish size across trials, log_10_ transformed initial mass and length were used as response variables in the same series of five analyses, this time computed with mixed‐model analyses of variance (ANOVAs) with treatment as the fixed effect and aquarium (replicate) as the random effect and conducting, where applicable, *post hoc* Tukey HSD pairwise comparisons if significant. As water temperature also influences fish physiological tolerance, a one‐way ANOVA series was conducted using log_10_ transformed initial water temperature within replicates as the response variable.

Finally, the impact of exposure type on fish behaviour was also investigated using a series of multinomial or binomial exact goodness‐of‐fit tests and one‐way ANOVAs. Goodness‐of‐fit tests were used to determine the differences in observed ASR percentages between treatments, with the amount of observed ASR averaged over the 10 observation periods conducted from 24 to 96 h post‐exposure. One‐way ANOVAs were used to test for differences in the three scored flume metrics across treatments, separately, with metrics first log_10_(*x* + 1) transformed. To investigate if there was any impact of fish size on flume test results, a series of one‐way ANOVAs were performed with log_10_ transformed fish mass and length used as response variables.

All one‐way and mixed‐model ANOVAs (nlme package) as well as the PCA were run in R version 2.13.0 (R Core Team, 2011) while all goodness‐of‐fit tests (XNomial package) were run in R version 3.6.1. For all tests, results are significant at ɑ < 0.05 and all presented means are ±1 standard deviation. Graphs were produced in R.

### 2.6 Ethical statement

Although ultimately obtained from a local bait supplier, a licence to collect fish for scientific purposes was issued by the Ontario Ministry of Natural Resources to conduct this study and investigate the consequences of exposing fish to dog waste water contamination (Licence No. 1093663). Applicable sections within the guidelines on the care and use of fish for research set forth by the Canadian Council on Animal Care (CCAC) were followed throughout the duration of this study, including animal acquisition, housing and handling. As per the CCAC all fish showing signs of acute distress in the form of loss of equilibrium or reduced ASR rate, as well as all fish at the conclusion of the experimental period, were euthanized using a lethal dose of clove oil.

## 3 RESULTS

### 3.1 Water quality and faecal analysis

**FIGURE 2:**
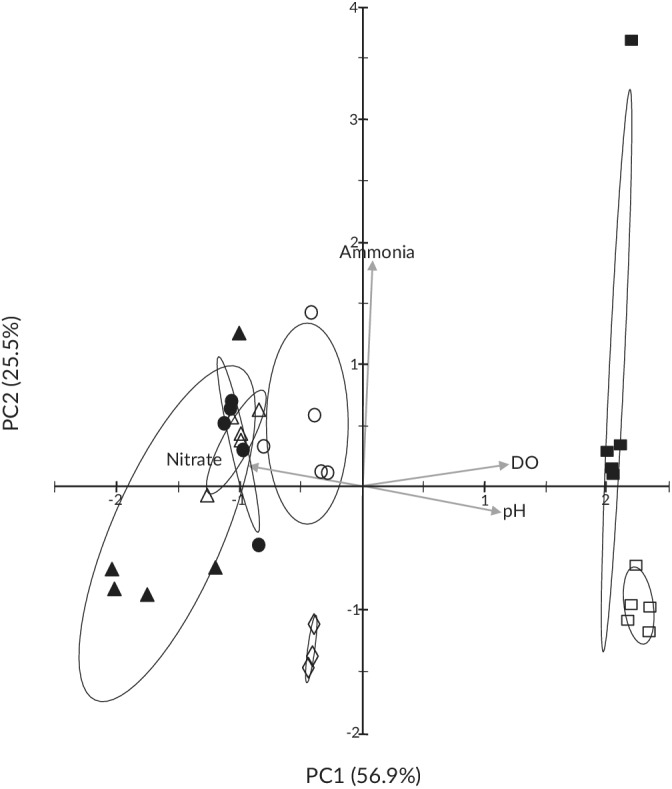
Biplot of principal component 1 (PC1) *vs*. PC2 showing trial scores and water quality vector loadings across treatments. F is fresh faeces, D dried faeces, A aerated trials, with numbers denoting the concentration level of the trials. Ellipses represent 68% confidence intervals. Treatment: 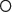, control×F; 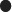, 1×F; 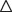, 2×F; 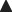, 4×F; 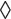, 4×D; 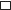, control×A; 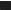, 4×A

The PCA grouped replicates and differentiated among treatments based on measured water quality parameters throughout the experimental period (Table 1 and Figure 2). Principal components 1 (PC1) and 2 (PC2) accounted for 82.4% of the total variance among trials, with PC1 mainly differentiating aerated trials, which were positively associated with DO and pH, from nonaerated trials, which were positively associated with nitrate levels. PC2 was largely driven by ammonia, low values differentiating dry faecal trials from fresh, and high values separating one of the 4×A replicates. Ammonia levels measured within this replicate were double those measured within the other four replicates, averaging 5.15 mg/l across the exposure period (range 3–6 mg/l) compared to an average of only 2.50 mg/l (range 1.75–3.2 mg/l), respectively. The greater degree of variation among 4×F trials compared to other concentrations was also due to the positive ammonia loading of a single replicate. While PC3 accounted for an additional 15.3% of the variation among trials, this axis was primarily associated with nitrate and only further differentiated 4×F trials from 1×F and 2×F trials.

**TABLE 1:** Average (±s.d.) water quality parameters across replicates for each treatment as a function of time post‐exposure

Water quality parameter	Time (h)	Treatment						
		Control×F	1×F	2×F	4×F	4×D	Control×A	4×A
pH	24	7.86 ± 0.07	6.46 ± 0.02	6.39 ± 0.02	7.58 ± 0.05	7.64 ± 0.01	8.31 ± 0.12	8.12 ± 0.04
	48	7.69 ± 0.09	7.60 ± 0.04	7.61 ± 0.02	7.69 ± 0.02	7.54 ± 0.03	8.40 ± 0.03	8.21 ± 0.03
	72	7.67 ± 0.03	7.60 ± 0.04	7.61 ± 0.03	7.67 ± 0.01	7.47 ± 0.06	8.41 ± 0.03	8.27 ± 0.03
	96	7.62 ± 0.03	7.65 ± 0.04	7.61 ± 0.03	7.69 ± 0.04	7.43 ± 0.10	8.39 ± 0.06	8.33 ± 0.04
DO (mg/l)	24	3.21 ± 0.22	2.17 ± 0.44	1.45 ± 0.35	0.00 ± 0.00	0.11 ± 0.02	7.35 ± 0.07	6.94 ± 0.09
	48	2.56 ± 0.21	2.08 ± 0.33	1.86 ± 0.44	0.00 ± 0.00	0.01 ± 0.01	7.44 ± 0.04	6.99 ± 0.06
	72	2.49 ± 0.34	1.77 ± 0.33	1.77 ± 0.14	0.00 ± 0.00	0.00 ± 0.00	7.20 ± 0.10	7.08 ± 0.05
	96	2.17 ± 0.19	1.26 ± 0.16	1.44 ± 0.15	0.03 ± 0.06	0.00 ± 0.00	7.47 ± 0.09	7.44 ± 0.12
Nitrate (mg/l)	24	26.00 ± 5.48	20.00 ± 0.00	28.00 ± 4.47	110.00 ± 0.00	6.33 ± 4.73	7.50 ± 0.00	7.50 ± 0.00
	48	24.00 ± 5.48	24.00 ± 5.48	30.00 ± 0.00	64.00 ± 50.92	0.33 ± 0.58	5.00 ± 0.00	5.00 ± 0.00
	72	35.00 ± 5.48	16.00 ± 5.48	10.00 ± 6.12	0.00 ± 0.00	0.00 ± 0.00	10.00 ± 3.54	10.00 ± 0.00
	96	35.00 ± 5.00	38.00 ± 4.47	23.00 ± 13.04	0.00 ± 0.00	0.00 ± 0.00	10.00 ± 0.00	10.00 ± 0.00
Ammonia (mg/l)	24	1.60 ± 0.32	2.14 ± 0.21	2.58 ± 0.48	1.40 ± 0.42	1.20 ± 0.10	2.10 ± 0.16	2.26 ± 0.43
	48	2.66 ± 0.68	2.32 ± 0.41	2.44 ± 0.34	2.00 ± 0.61	1.63 ± 0.23	2.20 ± 0.32	3.52 ± 1.17
	72	4.10 ± 0.82	2.10 ± 0.28	2.42 ± 0.29	2.10 ± 1.08	1.33 ± 0.15	1.00 ± 0.00	2.75 ± 1.82
	96	1.98 ± 0.61	3.28 ± 0.60	2.52 ± 0.22	2.20 ± 1.04	1.30 ± 0.17	1.10 ± 0.22	3.60 ± 1.34

*Note*. For treatments F is fresh faeces, D is dried faeces and A is aerated trials, with numbers denoting the concentration level. DO, dissolved oxygen.

Fresh faecal samples were 25.5% dry at the time of analysis, at which point they contained 11.11 mg/g total nitrogen (1.23%), 1.59 mg/g of which was from ammonia, 0.95% total phosphorus, 19.87 mg/g phosphate (2.19%) and 82×10^6^ total CFU per gram. Desiccated samples reduced in mass from 149.34 g when fresh to 56.46 g when dried. At the time of analysis they were 61% dry, contained 22.82 mg/g total nitrogen (2.51%), 0.77  mg/g of which was from ammonia, 2.38% total phosphorus, 49.67 mg/g phosphate (5.48%) and still 32×10^6^ CFU per gram.

### 3.2 Survival

**FIGURE 3:**
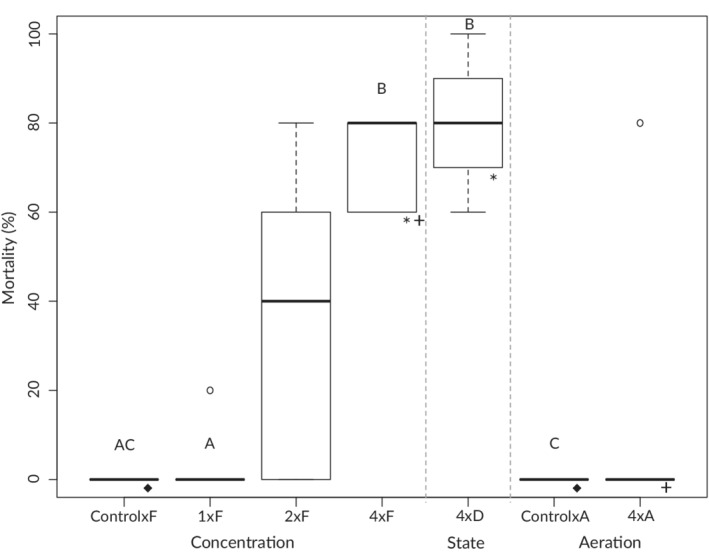
Observed final percentage mortality across experimental treatments. F is fresh faeces, D dried faeces and A aerated trials. All pairwise combinations on either side of the dotted lines were compared in addition to boxes with matching lower symbols using goodness‐of‐fit tests. Letters denote significance, with matching letters indicating no significant difference at *P* = 0.05. Boxplot whiskers are 1.5× the interquartile range

Exposure to dog faeces had a significant impact on fish survival. While only one individual died in 1×F trials, mortality averaged 36% in 2×F and 72% in 4×F trials, with both differing significantly from control×F (*P* < 0.001 and *P* < 0.001, respectively) which had no mortality (Figure 3). Time‐to‐mortality also decreased with exposure level, with the single 1×F mortality occurring at 24 h post‐exposure, the average time of mortality 17.9 h in 2×F (first death at 10.7 h) and 14.7 h in the 4×F treatment (first death at 8.2 h). Although there was no significant difference in the degree of mortality between 4×D (average 80%) and 4×F trials (*P* = 0.570), time‐to‐mortality was comparatively prolonged for dry trials (average 22.4 h, first death 12.1 h). Unsurprisingly, aeration significantly reduced the amount of observed mortality (average 16%, *P* < 0.001) and again prolonged the time‐to‐mortality (average 40.1 h, first 22 h) in 4×A relative to 4×F treatments.

**FIGURE 4:**
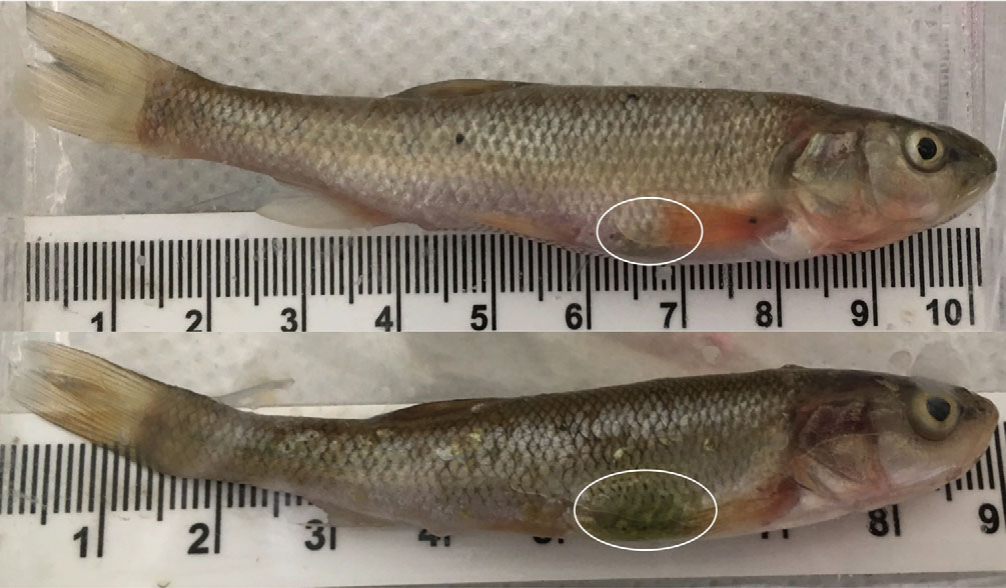
Examples of subdermal lesions of different severities observed on *Semotilus atromaculatus* individuals that died within 4×D (dried faeces) replicates

Unusual abdominal lesions and/or skin discolouration near the area over the stomach/pyloric cecae were observed in the majority of deceased fish (Figure 4), the severity of which increased with increasing post‐exposure time of death. While no lesions were observed on the one individual that died in 1×F trials, 89% of those that died in 2×F trials had lesions, primarily scored at level 2, and 72% of those that died in 4×F trials had lesions, primarily scored at level 1. Despite the prolonged time of death, lesions were only observed on 50% of those fish that died in 4×A trials, with 83% of those that died in 4×D trials having lesions, primarily scored at level 2. Only one individual which survived exposure was noted as also having unusual observable discolouration.

For the 12 aquaria across all treatments in which there was mixed mortality (one 1×F, three 2×F, five 4×F, one 4×A and two 4×D replicates), initial mass was significantly different (*W* = 736, *P* < 0.001) between those individuals that survived (3.81 ± 1.15 g) and those that died (6.32 ± 1.83 g). As there were no significant differences between treatments in fish mass at trial set‐up and little difference with respect to fish length (Table 2), size alone did not dictate survival or weight change independently of treatment exposure. Likewise, while temperature differed among treatments, pairwise comparisons (Table 2) did not correlate with observed mortality differences (*e.g*., control×F *vs*. 4×F). Average temperatures measured across treatments were well within both low and high critical, as well as growth‐specific temperature ranges for creek chub (McMahon, 1982; Smale & Rabeni, 1995).

**TABLE 2:** Average (±s.d.) fish initial mass, length and initial water temperatures across replicates for each treatment within aquaria as well as average (±s.d.) mass and length at the time of flume test participation

Test Measurement	Treatment						
	Control×F	1×F	2×F	4×F	4×D	Control×A	4×A
Aquaria							
Mass (g)	5.43 ± 1.46ad	4.51 ± 2.65a	4.74 ± 1.43a	5.88 ± 1.74 ace	6.95 ± 1.65ce	5.17 ± 1.53bd	5.53 ± 1.59b
Length (cm)	7.97 ± 0.74 ac	7.62 ± 1.22a	7.93 ± 0.86a	8.12 ± 0.79a	9.03 ± 0.91	8.47 ± 0.84bc	8.63 ± 0.80b
Temperature (°C)	23.29 ± 0.04a	24.28 ± 0.04b	24.29 ± 0.06b	23.24 ± 0.07a	18.06 ± 0.04	23.97 ± 0.05	24.03 ± 0.01
Flume							
Sample size (no. of fish)	10	7	7	7	5	8	8
Mass (g)	4.36 ± 0.87abch	7.40 ± 3.60ad	5.41 ± 3.46bde	3.71 ± 0.54cegi	3.97 ± 0.27 g	5.65 ± 4.21fh	5.02 ± 2.68fi
Length (cm)	7.56 ± 0.52abf	9.46 ± 1.07c	8.30 ± 1.37acd	7.27 ± 0.39bdg	8.34 ± 0.27	8.48 ± 2.19ef	8.38 ± 1.38eg

*Note*. All pairwise combinations of nonaerated fresh faeces treatments, aerated treatments, controls, 4×F and 4×D, and 4×F and 4×A were compared for significance with ANOVAs. Letters denote significance, with matching letters indicating no significant difference at *P* = 0.05. For treatments F is fresh faeces, D is dried faeces and A is aerated trials, with numbers denoting the concentration level.

### 3.3 Behavioural effects

**FIGURE 5:**
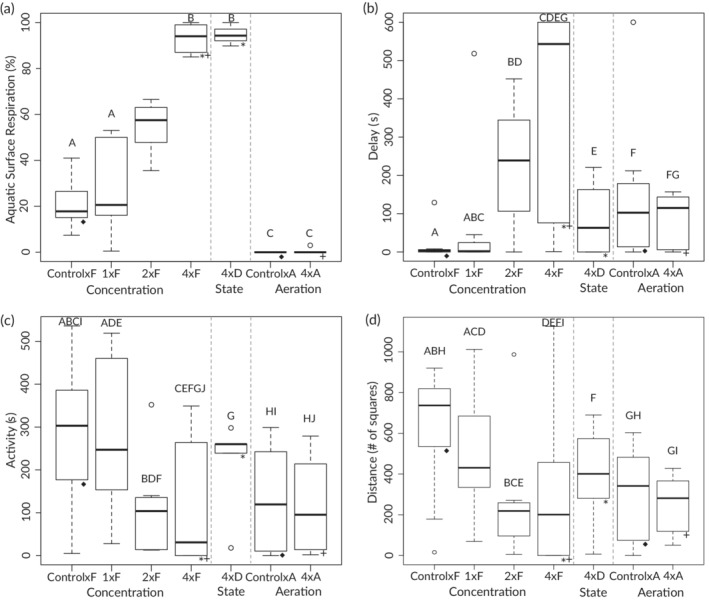
Observed average percentage aquatic surface respiration (a), swimming delay (b), activity (c) and distance (d) behavioural metrics for each faecal exposure treatment. F is fresh faeces, D dried faeces and A aerated trials. All pairwise combinations on either side of the dotted lines were compared in addition to boxes with matching lower symbols using either goodness‐of‐fit (a) or ANOVA (b–d) tests. Letters denote significance, with matching letters indicating no significant difference at *P* = 0.05 for each relevant test. Boxplot whiskers are 1.5× the interquartile range

Not only did exposure to dog faeces affect fish survival and weight, but behavioural changes were also observed. Again, 1×F trials did not (*P* = 0.480), while 2×F and 4×F trials did differ significantly from control levels (*P* < 0.001 and *P* < 0.001, respectively) with respect to ASR percentages (Figure 5a). Each increase in exposure above 1×F resulted in higher ASR rates, occurring an average of 21.5% of the observed time in control×F trials, 28% of the time in 1×F trials, 54% of the time in 2×F trials and increasing to a near constant 93% of the time in 4×F trials and 94.7% of the time in 4×D trials. As expected both aeration trials resulted in significantly less observed ASR (average 0% and 0.6%, *P* < 0.001 and *P* < 0.001, control×A and 4×A compared to ×F trials, respectively) and were correlated with reduced mortality.

Although analyses of flume‐derived swimming metrics indicated fewer significant differences among and between treatments, several behavioural patterns were still evident (Figure 5b–d). Among concentration trials, average delay increased while activity and distance travelled both decreased with increasing exposure levels. Only control×F and 2×F and control×F and 4×F trials differed significantly from each other with respect to delay (*P*_adj_ = 0.049 and *P*_adj_ = 0.025, respectively), with control×F and 4×F also differing with respect to distance (*P*_adj_ = 0.046). While the concentration ANOVA for activity was significant overall (*F*_(3,27)_ = 3.101, *P* = 0.043), there were no significant individual pairwise comparisons (*e.g*., control×F *vs*. 4×F, *P*_adj_ = 0.062). Large behavioural variation was observed within some treatments. In 4×F trials, for example, three of the seven individuals available to participate did not leave the flume acclimation area, which resulted in delay that varied from 1 to 600 s, activity that varied from 0 to 349 s and distance travelled that varied from 0 to 1125 squares. Only one other individual (control×A) remained within the flume acclimation area throughout the trial. Interestingly, 4×D exposed fish appeared less delayed, more active and travelled further than fish exposed to 4×F, despite experiencing similarly poor water quality/DO levels and high mortality and ASR percentages. Note that as with aquarium outcomes, differences were uncorrelated and exposure‐related variance in behavioural metrics was not considered to be a function of fish size (Table 2).

## 4 DISCUSSION

Exposure to realistic concentrations of dog faecal inputs caused significant size‐dependent mortality and reduced activity levels of creek chub, a widespread urban fish species. These findings indicate that uncollected dog waste, whether fresh or dried, has the potential to significantly impact the aquatic environment and the fish communities within.

### 4.1 Survival as a function of DO and bacterial loads

While none was observed in either of the control treatments, the amount of mortality increased significantly with increasing exposure concentration within nonaerated trials, regardless of whether the faecal samples were fresh or dried. Based on measured water quality parameters this was primarily a function of DO levels within trials, which were negatively correlated with the amount of faecal input and likely caused by increased BOD, with bacteria loads consuming DO during the decomposition of the organic carbon and ammonia within the dog waste itself (Penn *et al*., 2009). Although creek chub are considered tolerant of low DO and are able to survive for short periods within pools with 2.4 mg/l (McMahon, 1982) and have a critical concentration of 0.84 ± 0.08 mg/l (fish wt. 1–10.1 g, Smale & Rabeni, 1995), measured levels regularly fell below this value and reached near zero over the exposure period. Survival of individual fish was found to be size‐dependent, with survivors more likely to be creek chub with initially smaller body sizes. There is some debate as to whether low DO tolerance is actually related to fish body size (Nilsson & Östlund‐Nilsson, 2008; Tang *et al*., 2020). However, our results are similar to those of Robb and Abrahams (2003), who found that smaller fish were more tolerant of low DO environments with smaller individuals compensating by increasing ventilation frequency and haematocrit/haemoglobin concentration to a greater degree than larger individuals (see behavioural shifts below).

Dissolved oxygen alone, however, is unlikely to account for all aquaria results. For example, in the case of the 4×A replicate where DO would not have been a limiting factor, the 80% observed mortality was likely caused instead by elevated ammonia levels within the aquarium. Here, ammonia levels measured roughly double those within the other four replicates, averaging 5.15 mg/l across the exposure period (range 3.00–6.00 mg/l). Elevated ammonia levels in this range have been found to be negatively correlated with community response metrics, including creek chub abundance within channelized stream sites sampled across a gradient of water chemistry and water quality conditions (Smiley Jr. *et al*., 2009).

Interestingly, the majority of deceased individuals across exposure levels/types were found to have developed abnormal abdominal subdermal lesions. As environmentally derived faecal coliforms are known to be found in the intestinal tract of fish in areas with contaminated water and food (Geldreich & Clarke, 1966) it is likely that deceased fish either directly or indirectly consumed suspended or settled faecal particles prior to death. While we did not dissect these fish to determine the exact cause, the discolouration was clearly evident and in the exact same distinct and isolated location on each individual. The presence of DELT (deformity, eroded fin, lesion, tumour) anomalies has been found to be a good indicator of fish health and impaired water quality, particularly from waste pollution (Benejam *et al*., 2010; Simon & Burskey, 2016), with Yeom *et al*. (2007) also listing hypodermic haemorrhages as one of the DELTs found in association with fish exposed to wastewater effluent.

### 4.2 Behavioural shifts and population‐level implications

Three ways that free‐swimming fish can compensate for low DO availability are to increase ventilation rate, perform ASR and decrease oxygen demand by down‐regulating metabolism and reducing energy expenditure (Nilsson & Östlund‐Nilsson, 2008; Pollock *et al*., 2007). Here creek chub ASR rates were found to increase with increasing exposure concentration within nonaerated water and reached a near‐constant 93%–95% of the time within 4×F and 4×D trials. While creek chub ASR had been previously reported in Gee *et al*. (1978), no test of the change in creek chub swimming behaviour as a result of low DO (Chapman & Mckenzie, 2009; Domenici *et al*., 2012) or pollutant/contaminant exposure (Little & Finger, 1990; Melvin & Wilson, 2013) was found within reviews on these topics. Here, average delay time increased while time of activity and distance travelled both decreased with increasing concentration of nonaerated fresh faecal samples. This reduction in swimming activity is consistent with the energy conservation hypothesis, and has been suggested as an adaptation for fish that commonly encounter low DO environments and, similar to creek chub, tend to be demersal and relatively sedentary (Chapman & Mckenzie, 2009; Domenici *et al*., 2012). As with mortality, such behavioural changes were likely not due to DO alone, with the lethargy and lack of movement exhibited also indicative of a potential metabolic immune response (Bonneaud *et al*., 2016). Indeed relative to 4×F trials, fish within 4×D trials experienced similar low DO and exhibited similar ASR rates, but were less delayed, more active and travelled a greater distance within flume tests, and were even more active compared to 4×A trials. As 4×D samples contained reduced bacterial loads, the less depressed swimming motivation may have been due to a lesser required immune response within surviving fish compared to that necessary among 4×F or even 4×A survivors.

Such observed behavioural changes come at a physiological cost and, along with the observed size‐dependent mortality, may have the potential to have large impacts on wild fish populations by altering population size‐class structure and dynamics. Indeed, larger size‐classes of fish have been found to be missing from populations sampled within contaminated waters, and older creek chub, for example, were missing from sites as a result of PCB contamination in Henshel *et al*. (2005). While it is possible that the decreased overall abundance within populations was due to emigration of individuals from chronically polluted areas in search of more suitable habitat, activity here was down‐regulated and larger fish may instead experience direct mortality under some dog faeces/runoff conditions. Smaller individuals remaining in populations within chronically polluted waters may not experience such immediate mortality, however the behavioural shifts observed here as a function of pollutant exposure may result in increased vulnerability to predators (Kramer, 1987) both due to increased visibility (ASR; Chapman & Mckenzie, 2009) and decreased escape‐responses (swimming activity/speed; Domenici *et al*., 2012). By extension, fish may experience reduced foraging efficiency and thereby growth as well as reduced reproductive development and success, ultimately leaving surviving populations with overall reduced health and fitness (Bonansea *et al*., 2016; Bonneaud *et al*., 2016; Kramer, 1987; Melvin & Wilson, 2013; Pollock *et al*., 2007; Scott & Sloman, 2004; Vieira *et al*., 2009).

### 4.3 Real‐world relevance

While creek chub are not the most urban pollution tolerant species (van den Hurk *et al*., 2017) they are one of the most widespread stream minnows in eastern North America (Dubé *et al*., 2006) and are found across a range of other contaminant gradients. As expected, populations found in polluted waters are less abundant and part of less diverse fish assemblages (Katz & Gaufin, 1953; Simon *et al*., 2013; Stair *et al*., 1984). Compared to Cyprinids, Centrarchid species are more pollution (van den Hurk *et al*., 2017) and nitrite (Lewis & Morris, 1986) tolerant while Salmonid species are more nitrite sensitive (Lewis & Morris, 1986; Russo *et al*., 1974) and less hypoxia tolerant (Gee *et al*., 1978; Nilsson & Östlund‐Nilsson, 2008). As species from these families would potentially present lesser and more acute responses to exposure than those found within this study, future work should investigate the responses of other species and their particular behavioural shifts, such as changes in schooling, homing ability, spawning attempts and parental care, all of which are also influenced by environmental pollutants (Scott & Sloman, 2004). Determining the mechanistic pathways and causal mechanisms underlying the mortality and behavioural shifts observed here was outside the scope of this paper. As this was the first investigation of the potential effects of uncollected dog waste on urban fish populations, it serves as a foundation for future work to build on the basic observations provided here and to investigate the metabolic pathways and enzymatic activity alterations by which such changes are mediated (*e.g*., van den Hurk *et al*., 2017; Vieira *et al*., 2009).

The real‐world relevance of the concentrations of dog faeces used in this study can be demonstrated with the following example. Using the median urban pond size in the United Kingdom of 293.4 m^2^ and a maximum depth of 1.4 m (Glendhill *et al*., Gledhill *et al*., 2008), ~18 kg of dog faeces would be required to achieve the 1× concentration (0.092 g/l) used in this study. This equates to roughly 41 small dogs/day or 13 larger dogs/day over 3 days (Ellis, 2004; Oates *et al*., 2017). These estimates drop when considering only the volume of water within a 5 m littoral zone around the pond (31 small dogs/day over 3 days or 10 large dogs/day over 3 days). Of course the nature of the input need not be direct as urban water bodies act as receiving basins for large drainage areas, and Ervin *et al*. (2014) showed that even small upstream inputs can alter downstream water quality. The overall amount of input required to reach a particular concentration itself depends on several factors. Fewer deposits would be required within smaller water bodies, such as those that may be found in more highly urbanized and densely populated areas or within urban creeks and streams with isolated pools that form during drought and low summer flow conditions. Moving forward, it is worth investigating the impacts of uncollected dog waste on populations within normal to high‐flow streams and at beaches, habitats that not only provide more continuous inputs from exchanging waters but also expose populations to additional physical and physiological demands and stressors. It would also be worth investigating the potential interactive effects of faecal contamination with other contaminants present within urban waterways.

While to some members of the public uncollected dog waste may not seem like a relevant problem, to others the impact it has on public spaces is already well known. Every year hundreds of kilograms of dried dog waste (*e.g*., Walker & Garfield, 2008) is often found uncollected in urban parks and along trails and beaches, translating to potentially thousands of kilograms of faecal loading per year (Oates *et al*., 2017). As at least a partial result public beaches, ponds and streams increasingly test higher for faecal coliform bacteria than the standard safe levels for human recreational use (Oates *et al*., 2017; Serrano & DeLorenzo, 2008) and toxic algal blooms containing compounds such as microcystins and botulinum have become a more frequent occurrence. Despite the potentially direct ecological and economic repercussions (Carpenter *et al*., 1998), this study showed for the first time the impact that the global dog waste management problem can have on aquatic communities. The effects demonstrated here are likely to not only be more severe for less pollution‐tolerant species, but are likely to become more apparent for many urban fish populations and other aquatic communities as the dog population, the amount of urbanization, the relative prevalence of impervious surfaces and climate change all continue to increase.

## AUTHOR CONTRIBUTIONS

Both authors developed the experimental design and were responsible for both the data analysis and manuscript preparation.
